# Epistaxis first-aid management: a needs assessment among healthcare providers

**DOI:** 10.1186/s40463-020-00485-8

**Published:** 2021-02-11

**Authors:** Leigh Sowerby, Chandheeb Rajakumar, Matthew Davis, Brian Rotenberg

**Affiliations:** 1 Department of Otolaryngology – Head & Neck Surgery, Schulich School of Medicine & Dentistry, Western University, St. Joseph’s Healthcare, 268 Grosvenor Street, London, ON N6A 4V2 Canada; 2 Department of Surgery, Division of Otolaryngology – Head & Neck Surgery, University of British Columbia, Vancouver, BC Canada; 3 Division of Emergency Medicine, Department of Medicine, Schulich School of Medicine & Dentistry, Western University, London, Ontario Canada

**Keywords:** Urgent care, Continuing medical education, First aid, Epistaxis, Healthcare professionals, Compression, Survey study

## Abstract

**Purpose:**

To perform a needs assessment of epistaxis first-aid measures practiced by family physicians and Emergency Department (ED) staff in London, Ontario, Canada.

**Methods:**

Paper-based multiple-choice questionnaires were distributed to participants. Participant recruitment was conducted in two parts: 1) 28 Emergency Medicine (EM) attending physicians, 21 resident physicians training in the ED, and 26 ED nurses were surveyed while on duty in the ED; 2) 27 family physicians providing walk-in or urgent care and attending a continuing medical education (CME) event were also surveyed. Respondents were asked to identify where to apply compression to the nose and how patients should be positioned during acute epistaxis.

**Results:**

Regarding where to apply compression, 19% of family physicians, 43% of EM physicians, 24% of residents, and 8% of ED nurses responded correctly. Regarding positioning, all groups responded similarly with 54–62% responding correctly. Twenty-one percent of emergency physicians, 19% of residents, 11% of family physicians, and 4% of nurses responded correctly to both questions.

**Conclusions:**

Most family physicians, EM attending physicians, ED nurses, and residents could not correctly identify basic first-aid measures for acute epistaxis. This study identifies an area where knowledge is lacking and the potential for improvement in patient management and education.

**Grapical Abstract:**

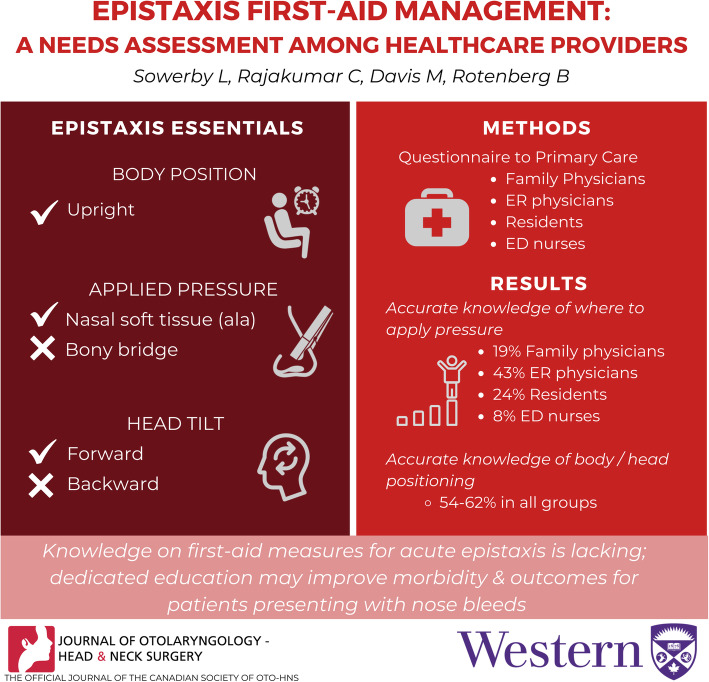

## Introduction

Epistaxis is a common problem and is potentially life-threatening. The lifetime incidence of epistaxis in the general population is 60% [1]. Of those having epistaxis, 10% are treated by a physician at some point. Some evidence has been published suggesting that Emergency Department (ED) staff (physicians and nurses) are using inappropriate first-aid measures in initial epistaxis management [2]. Over the last 20 years, there has been no indication that practice patterns are changing.

Basic first-aid measures for epistaxis should be known by all those working in the ED and those involved in community-based care [3]. In short, sitting the patient up and tilting the head forward can protect the airway and pinching the nasal ala against the septum for 10 to 15 min applies pressure to Kiesselbach’s plexus, the source of bleeding in 95% of cases [4]. When correctly performed, these procedures are a simple and effective first-aid measure to stop an active nosebleed in most cases [5]. Nevertheless, many pervasive myths still exist surround epistaxis first-aid. More common misconceptions include tilting the head backwards and applying pressure to the nasal bones or the rhinion. Though these misconceptions may be common among the general public, it would raise concern if health care providers were propagating these inappropriate treatments. Epistaxis first-aid management is not only described in Otolaryngology (Oto-HNS), EM, and Family Medicine texts and journal articles but is also part of basic life support courses [4]. The objective of this study is to assess knowledge of first-aid epistaxis management exists in primary care physicians, EM physicians, ED nurses and residents rotating through Emergency Medicine.

## Methods

### Instrument and outcome measures

**Fig. 1:**
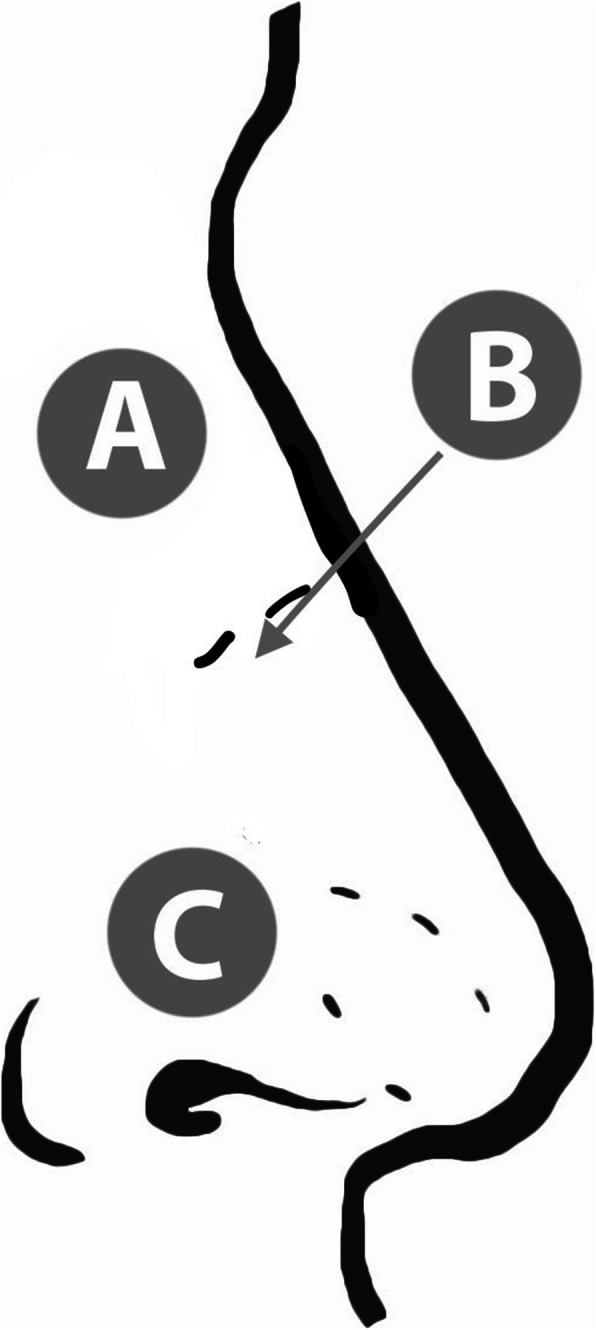
Nose diagram for location for pressure application

A paper-based multiple-choice survey was prepared to assess participants’ familiarity with epistaxis first-aid measures and administered after formal Research Ethics Board approval from Western University (REB#104577). Through forced-choice questions, participants were asked where to apply pressure on a picture of a nose (the nasal bones, mid-dorsum/rhinion, or ala – Fig. 1) and how the patient should be positioned (head neutral, tilted forward, or backward). For the location of compression, a diagram was provided to ensure participants understood the available choices. Pressure applied to the ala with head tilted forward was considered correct. Participants were also asked for their degree of confidence for each response (unsure, somewhat confident, or very confident). Questions and illustrations were initially validated among Otolaryngology residents at Western University to ensure accuracy of the questionnaire – the resident performance was uniformly accurate on the questionnaire.

The proportion of patients who answered incorrectly and stated that they were “very confident” about their response was also determined. The distribution of information sources that respondents from each group cited was also determined. Descriptive statistics were conducted using SPSS Statistics Version 22.0 software (IBM Corp., Armonk, New York) and the results are expressed as counts and percentage.

### Study participants

Groups surveyed included practicing family doctors, EM attending physicians, ED nurses, and residents of various training levels and from various training programs currently on EM rotations. Surveys for those working in emergency medicine was a convenience sample at the time of Oto-HNS consultation for a different matter until the sample size was reached. EM attending physicians were all fully licenced and held faculty positions with Western University. ED nurses were included as they are often the first healthcare workers to assess and care for patients in the ED. All EM attending physicians and ED nurses were practicing in one of three urban EDs, which serves a population of 380,000 people and is the tertiary care centre for a catchment population of over 1 million. Primary care practitioners providing walk-in or urgent care and attending a continuing medical education (CME) event were also surveyed. All participants completed their survey anonymously and independently at the time of consent to participation.

## Results

In total, 102 completed surveys were included for analysis. The respondents were comprised of 28 EM attending physicians (43% of total faculty), 26 ED nurses, 21 residents on EM rotations, and 27 family physicians.

**Fig. 2:**
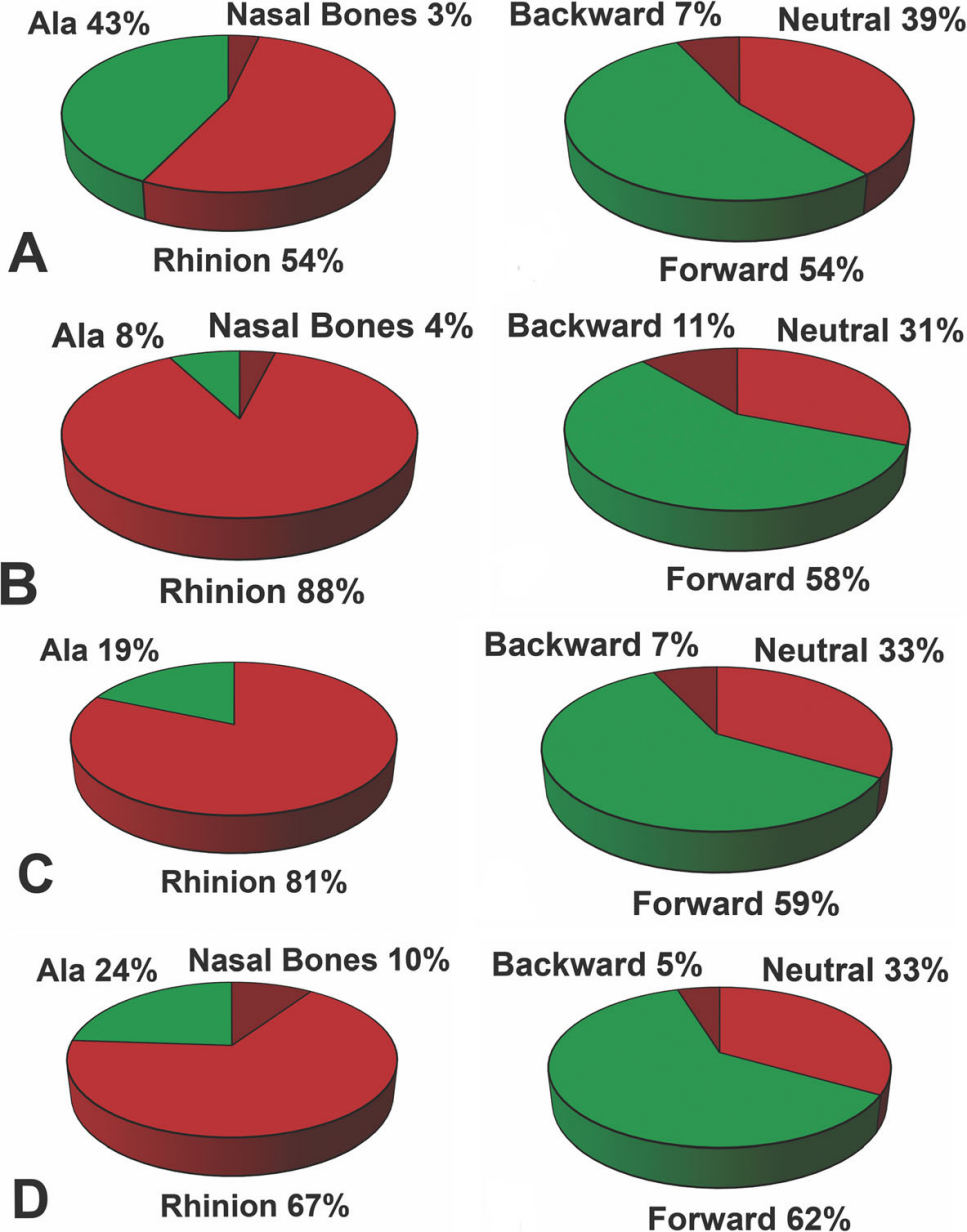
**a** EM attending physician, **b** EM nurse, **c** Resident on EM rotation, **d** Primary Care Physician

A minority of each group responded correctly when asked where to apply compression and just over half of respondents correctly positioned the patient’s head (Fig. 2). When assessing the responses to both questions together, only 21% of EM attending physicians, 19% of residents, 11% of primary care practitioners, and 4% of nurses responded correctly. A large proportion of EM physicians and primary care practitioners that responded incorrectly to either of the above questions stated they were “very confident” about their response (Table 1).

**Table 1:** Degree of Confidence among Incorrect Responders to Nasal Compression and Patient Positioning questions during Epistaxis

Group	Question with Number of Incorrect Responders (n)	Degree of Confidence		
		Unsure *n (%)*	Somewhat Confident *n (%)*	Very Confident *n (%)*
EM Attending Physicians	Location of Compression (16)	1 (6)	3 (19)	12 (75)
	Patient Position (13)	0	4 (31)	9 (69)
Emergency Department Nurses	Location of Compression (24)	1 (4)	16 (67)	7 (29)
	Patient Position (11)	2 (18)	8 (72)	1 (9)
Primary Care Physicians	Location of Compression (22)	0	11 (50)	11 (50)
	Patient Position (11)	2 (18)	4 (36)	5 (46)
Residents	Location of Compression (16)	1 (6)	13 (81)	2 (13)
	Patient Position (8)	3 (38)	5 (63)	0

Regarding the source of participants’ knowledge of epistaxis first-aid management, the majority of EM and family physicians cited both professional training and clinical experience, while most nurses cited clinical experience alone.

## Discussion

The present study shows that the general knowledge of epistaxis first-aid was poor in this population of health care providers. Furthermore, a large proportion of both family physicians and EM attending physicians replied that they were “very confident” when providing their incorrect responses. This suggests that these physicians are not simply uninformed on this subject, but may hold deeply established misconceptions on how epistaxis should initially be managed.

Unfortunately, these findings are not unexpected. In a recent survey of the junior physicians at 50 different United Kingdom (UK) EDs, 42% reported having no formal teaching on epistaxis management and 38% reported that the topic was covered in less than 15 min [6]. A study from the mid-1990s surveyed 115 ED staff members at a major UK teaching hospital on where to apply pressure to the nose during acute epistaxis [2]. They found that 33% of participants responded correctly. However, their respondents only included 25 physicians and included non-clinical as well as clinical ED staff.

The general public’s poor knowledge on this subject highlight the importance of healthcare team members as an accurate source of information. The problem also appears to be global in breadth. Strachan surveyed nearly 500 patients in the United Kingdom presenting for a variety of reasons on epistaxis first-aid. Only 11% of patients correctly responded to where to apply pressure and how to position one’s head [7]. A study by Alyahya et al. of medical students in Saudi Arabia found that only 41% identified the correct area for application of pressure on the nasal ala, with the majority reporting this knowledge being self-taught [8]. Another study in the United Kingdom surveyed 210 ED nurses in a similar fashion to our study. They found that only 12% of advanced nurse practitioners and 14% of staff nurses were able to answer all three questions accurately for position, pressure location and duration [9].

There is evidence that intervention in education for first-aid management of epistaxis helps. In a study of patients being seen by OtoHNS for epistaxis, all had seen a physician for epistaxis prior to seeing the Otolaryngologist, but less than half had received any advice about first-aid management – and those that did, were more likely to describe correct measures [10]. Eze et al. surveyed EM physicians on their practices advising patients and performed a pre−/post-intervention chart review [11]. EM physicians were given a presentation and leaflets on epistaxis first-aid management by the Otolaryngology department. Initially, 17% of patients returned to the ED with recurrent epistaxis. In the second phase of study, after education and provision of leaflets, only 8% returned to care with recurrent epistaxis. The 2020 American Academy of Otolaryngology clinical practice guidelines for epistaxis also identify addressing this knowledge gap as a quality improvement opportunity [12]. Ambiguity regarding these instructions unfortunately persists - a recent publication in the Journal of Family Practice reviewing epistaxis management states to “apply digital pressure at the cartilaginous part of the nose” and the importance of these instructions were not stressed [13]. To better improve patient education, we need to ensure teaching and education of healthcare workers is explicit both in instruction and importance.

Though our present study sheds light on a knowledge deficit in the surveyed health care providers, there are limitations to this study that warrant discussion. This survey was a single-center study and therefore, has inherent limitations to its generalizability. A forced choice survey may not capture what EM attending physicians specifically state to the patient as advice or the subtleties of where they compress the nose.

## Conclusion

The majority of family physicians, EM attending physicians, EM nurses, and residents on EM rotations surveyed in this study at a single centre incorrectly identified first-aid measures for acute epistaxis. Dedicated teaching on this topic can hopefully address and improve the knowledge deficit identified by this study, and subsequently improve patient care and safety.

## Data Availability

All data generated or analysed during this study are included in this published article.
